# R. W. Varney

**Published:** 1872-05

**Authors:** J. C. Wyman

**Affiliations:** Sec’y.


### R. W. Varney.

The following resolutions were passed by the Brooklyn Dental Society on the death of Dr. Varney, who died at Savannah, Ga., on the morning of April 12, 1872.

Whereas, It has pleased Almighty God to remove from our midst our late and much esteemed brother R. W. Varney, M. D., of New York City; and, whereas by this sudden and unexpected visitation of Divine Providence, we are deprived of the services and wise counsel of our late associate, we deem it proper and just to give expression of our feelings on the occasion of this bereavement; therefore, be it

*Resolved*, That the Society have heard with unfeigned sorrow of the demise of our brother, R. W. Varney, M. D., for whom we hoped a long and useful career in the practice of his chosen and well-loved calling.

*Resolved*, That in his death our profession has met with a severe loss, as his attainments, considering the shortness of his professional career, have been remarkable, and his record, as a member of the dental profession, having few if any parallels. A noble spirit of integrity pervaded all his professional efforts. The works of his hands we have many times looked upon with the highest admiration. Words cannot fully record his eulogy, and the influence of his character will long remain with us as a beacon light, urging us to emulate his example in making high professional attainments.

*Resolved*, That we deeply sympathize with his sorrowing widow in her severe affliction, and commend her to the keeping of Him who "was a man of sorrows and acquainted with grief."

*Resolved*, That we bow with submission to the divine will, and believe, as our brother, Dr. Varney, has often said, "that our Heavenly Father doeth all things well."

*Resolved*, That we will attend the funeral wearing the usual badge of mourning, and that the Secretary of the Society be requested to forward a copy of these resolutions to Mrs. Varney.

J. C. WYMAN, Sec'y.

